# Modification of Thiol-Ene Ionogels with Octakis(methacryloxypropyl) Silsesquioxane

**DOI:** 10.3390/polym13030385

**Published:** 2021-01-26

**Authors:** Aneta Lewandowska, Piotr Gajewski, Katarzyna Szcześniak, Mariola Sadej, Piotr Patelski, Agnieszka Marcinkowska

**Affiliations:** Department of Polymers, Faculty of Chemical Technology, Institute of Chemical Technology and Engineering, Poznan University of Technology, Berdychowo 4, 60-965 Poznan, Poland; aneta.b.lewandowska@doctorate.put.poznan.pl (A.L.); piotr.gajewski@put.poznan.pl (P.G.); katarzyna.szczesniak@put.poznan.pl (K.S.); mariola.sadej@put.poznan.pl (M.S.); piotr.r.patelski@student.put.poznan.pl (P.P.)

**Keywords:** ionogel, thiol-ene photopolymerization, POSS, electrochemical conductivity

## Abstract

The effect of polyhedral oligomeric silsesquioxane (POSS) on the synthesis and properties of hybrid organic–inorganic ionogels was investigated using octakis(methacryloxypropyl) silsesquioxane (methacryl-POSS). Ionogels were prepared in situ by thiol-ene photopolymerization of triallyl isocyanurate with pentaerythritol tetrakis(3-mercaptopropionate) in a mixture of imidazolium ionic liquid 1-ethyl-3-methylimidazolium bis(trifluoromethylsulfonyl)imide (EMImNTf_2_) and propylene carbonate (PC). Investigations included the kinetics of hybrid materials formation and selected physical and mechanical properties. The disadvantage of ionogels without the methacryl-POSS modifier is leakage and insufficient mechanical properties. Modifying the thiol-ene matrix by the addition of methacryl-POSS made it possible to obtain non-leaking ionogels with improved mechanical and conductive properties. The steric hindrance of POSS cages and high-density network formation played important roles in ionogel synthesis: decrease of polymerization rate (with almost no effect on conversion), as well as dimensions of the formed polymer spheres during dispersion polymerization (highly cross-linked polymer has poorer solubility in polymerizing medium at a similar conversion, and nucleation begins at lower conversion), an increase of glass transition temperature and puncture strength. Hybrid ionogels with high ionic conductivity in the range of 4.0–5.1 mS∙cm^−1^ with the maximum parameter for 1.5 wt.% addition of the methacryl-POSS were obtained, which can be associated with ion-pair dissociations in ionic liquid clusters caused by methacryl-POSS.

## 1. Introduction

Hybrid organic–inorganic ionogels are considered to be a new class of hybrid material, containing solid matrix (organic–inorganic nanoparticles in the polymer) and physically entrapped (immobilized) ionic liquid (IL) that keep their unique properties. These materials can be used as membranes or gel polymer electrolytes (GPEs) to produce electrochemical devices (e.g., batteries, fuel cells, supercapacitors). Hybrid ionogels can be obtained by an in situ process based on the polymerization of monomers and fillers dissolved/dispersed in ILs. Classical radical photopolymerization involving (meth)acrylates, represents a valuable approach that exhibits unique spatial, temporal control by means of a chain-growth mechanism, low-temperature cure, and energy efficiency. This process is widely used for a range of applications (coatings, inks, adhesives, dental materials, photolithographic processes) [1,2,3,4,5,6]. Photopolymerization of (meth)acrylates has several drawbacks, including inhibition of the polymerization by oxygen, the presence of unreacted monomer after curing following cure (in the areas not accessible to light), significant shrinkage that occurs during the polymerization process, and the formation of highly heterogeneous polymer networks [7,8,9,10,11].

The application of thiol-ene reaction effectively combines the classical benefits of click reactions with the advantages of a photoinitiated process, resulting in an excellent method of chemical synthesis and production of tailorable materials [1,12]. In comparison to (meth)acrylate photopolymerization, the process with the addition of thiol proceeds with reduced or no oxygen inhibition (which enables UV curing without the need for an inert gas atmosphere), delays the gel point conversion, reduces shrinkage stress that arises during the polymerization and forms ideal, homogeneous polymer network with narrow glass transition regions [1,10]. 

In our continuing investigations on the preparation and properties of photocurable ionogels with a thiol-ene matrix (based on triallyl ene and trithiol) [11], we undertook research concerning special hybrid nanofillers, i.e., polyhedral oligomeric silsesquioxanes (POSS). POSS is described by the general formula (RSiO_1.5_)_n_ or T_n_ (where n is the number of repeat units and T is RSiO_1.5_) wherein the substituent R can be reactive or nonreactive in the polymerization process [13,14]. This type of modifiers with a hybrid organic–inorganic nature and well-defined structure (hybrid character obtained by covalent organic–inorganic links at the nanoscale) has become very interesting due to the scope of their possible application [14]. POSS usually improves thermal and dimensional stability, surface hardness, and other mechanical properties of polymers by acting as nanofiller [15,16,17]. Moreover, this modifier usually changes the polymer chain topology and can provide the additional free volume to the polymer matrix owing to a steric effect, which results in high mobility of the polymer chain and decrease its glass transition temperature [15,18], although the opposite effect of POSS addition was also reported [19]. When POSS molecules aggregate it leads to introducing topological constraints and an increase in Tg. These limitations are associated with the steric hindrance of the rigid domains of POSS to the movement of the polymer chain segments [19].

POSS containing thiol groups was introduced into acrylated castor oil to develop a novel photocurable hybrid material with reduced surface energy and better thermal stability [20]. Fang et al. [21] reported that inorganic–organic hybrid fibers composed of a thiol-ene cross-linked network containing POSS molecules, have been successfully prepared directly from monomeric liquids by simultaneous centrifugal spinning and UV initiated thiol-ene process. The POSS containing thiol-ene fibers exhibited enhanced thermomechanical properties compared to purely organic analogs (4 times increase in modulus after thermal treatment at 100 °C).

Despite many works on polymer/POSS materials, only a few papers focused on the kinetics of the photocuring process. Usually, a small amount of POSS can substantially enhance the photopolymerization rate, both in the case of classical radical-based photopolymerization [18,22] and the thiol-ene system [20]. What is important, there are no reports on the photocuring behavior of systems thiol-en-POSS in ILs. The addition of POSS into the photocurable formulation can probably affect the mechanism of thiol-ene polymerization in ILs.

Nanofillers are incorporated into ionogels mainly to improve their thermal and mechanical properties. The detailed review of the fabrication of hybrid materials with special attention paid to polymer-based ionogels is presented in [23]. The incorporation of inorganic nanoparticles (mainly modified silica) into the ionogels can counterbalance the plasticizing effect of flexible thioether linkages and/or high IL loadings while preserving ionogel flexibility. Sometimes, the addition of a hybrid modifier to GPEs enhances its ionic conductivity due to specific interactions between the surface groups of nanofillers and ILs, occurring at the IL/filler interface, which promotes ion-pair dissociations in IL clusters [24]. GPEs with tunable network structures are prepared by a facile one-pot reaction of polyhedral oligomeric silsesquioxane [25]. POSS provides mechanical strength for a solid polymer electrolyte (SPE), while its small particle size ensures a uniform structure of SPE, in addition, its well-defined surface chemistry allows the architecture of the SPE network to be easily tuned. The POSS network-based SPEs exhibit excellent conductivity, mechanical properties, lithium dendrite growth resistance, and improved cycling stability [25]. Composite electrolytes containing an organic/inorganic hybrid star-shaped polymer, P(PEGMA-r-MA-POSS), PEG-functionalized POSS, and LiNTf_2_ in various blended compositions were also studied by Kim et al. [15,26]. 

GPEs can be also applied in electrochemical capacitors (EC) where plays the role of separator and electrolyte simultaneously. The most important advantages of using GPE are (i) preservation against the electrolyte leakage from ECs even in the case of its damage and (ii) simplification and time saving of ECs fabrication processes. Time saving is particularly noticeable if GPE is produced by the photopolymerization process [27,28]. However, the application of GPE in electrochemical devices requires their high conductivity and mechanical stability. Only GPE that meets both requirements may be applied in ECs.

The thiol-methacrylate system uniquely exhibits a mixed step-chain growth polymerization regime that combines the step-growth thiol-ene reaction and chain free-radical polymerization. This provides a simple route to prepare a novel POSS-based polymer. The effect of ILs and POSS on thiol-ene polymerization can differ from that observed for (meth)acrylate polymerization, because of different polymerization mechanism. The incorporation of methacryloxy functionalized-POSS derivatives is expected to enhance the mechanical and conductive properties of thiol–ene-POSS ionogels.

In studies presented in this paper to reduce the costs of ionogel, we decided to use for the synthesis a mixture of IL with propylene carbonate instead of the neat IL. As monomers triallyl isocyanurate (TATT) and pentaerythritol tetrakis(3-mercaptopropionate) (PETMP) were used. The obtained poly(TATT + PETMP) ionogel with the solvent mixture has a major disadvantage of leakage. To prevent this problem, we decided to modify the system with the addition of methacryl-POSS, which should also improve the mechanical properties of the ionogel. The aim of the study was therefore the synthesis of hybrid ionogels modified with the addition of POSS by the thiol-ene photopolymerization in the IL + PC mixture. Moreover, the study of the POSS influence on the kinetics of the reaction and the properties of the obtained ionogels.

## 2. Materials and Methods

### 2.1. Materials

Monomers: pentaerythritol tetrakis(3-mercaptopropionate) (PETMP), purity ≥95%, 1,3,5-triallyl-1,3,5-triazine-2,4,6(1H,3H,5H)-trione (TATT), purity 98%. Monomers were delivered by Sigma–Aldrich (St. Louis, MO, USA). Octakis(methacryloxypropyl) silsesquioxane (8M-POSS, methacryl-POSS), was provided by Hybrid Plastics (Hattiesburg, MS, USA). 1-ethyl-3-methylimidazolium bis(trifluoromethylsulfonyl)imide (IL, EMImNTf_2_), purity 99% was delivered by Solvionic (Toulouse, France). Propylene carbonate (PC) anhydrous, purity 99.7%, was purchased from Sigma–Aldrich (St. Louis, MO, USA). The photoinitiator, 2,2-dimethoxy-2-phenylacetophenone (DMPA) was also supplied by Sigma–Aldrich (St. Louis, MO, USA). 

### 2.2. Methods 

#### 2.2.1. Isothermal Differential Scanning Photocalorimetry (Photo-DSC)

The kinetics of photopolymerization of the thiol-ene compositions modified with methacryl-POSS (with or without solvents mixture) was determined by using isothermal differential scanning calorimetry (DSC, Pyris6 instrument, Perkin–Elmer, Waltham, MS, USA). The DSC apparatus was equipped with a lid especially designed for photopolymerization measurements. The 2 mg samples were polymerized in open aluminum pans (diameter 6.6 mm) under isothermal conditions at 25 °C. All reactions were performed under high-purity argon atmosphere (<0.0005% of O_2_). The polymerizations were initiated with the UV light (LED lamp Hammamatsu, LC−1, *λ_max_*= 365 nm, *I*_0_ = 1 mW∙cm^−2^, Iwata City, Japan). All the experiments were conducted at least in triplicate. The reproducibility of the kinetic results was about ±3%.

#### 2.2.2. Solvatochromic Solvent Parameters

**Figure 1:**
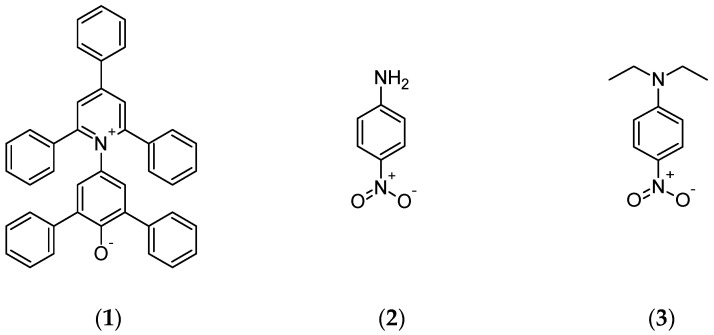
The structures of solvatochromic dyes: (**1**) Reichardt’s dye, (**2**) 4-Nitroaniline, (**3**) *N,N*-diethyl-4-nitroaniline.

4-Nitroaniline, purity > 99%; *N,N*-Diethyl-4-nitroaniline, purity 98% and Reichardt’s dye, purity 90% (Figure 1) were used to determine solvatochromic parameters: Reichardt’s empirical ET(30) polarity parameter, normalized polarity parameter ETN, empirical Kamlet–Taft polarity parameters: π* (dipolarity/polarizability), *α* (hydrogen bond donating ability), and *β* (hydrogen bond accepting ability). All dyes were delivered by Sigma–Aldrich (St. Louis, MO, USA). Solvatochromic dyes were dissolved in anhydrous methanol in the concentration of 5 × 10^−3^ M. The dye concentrate was added to the IL or solvent in such an amount that the maximum absorption band was in the range of 0.4 to 0.5. The mixture was homogenized and then, the methanol was evaporated at 40 °C under reduced pressure. The absorption spectrum was measured for each dye by spectrophotometer Jasco UV-530 (Tokyo, Japan). Measurements were carried out at room temperature in a quartz cuvette with a light path length of 1 mm in the measuring range 200–800 nm. The solvatochromic parameters were calculated according to Equations (1)–(5) and the methods described in the articles [29,30,31,32,33]:

(1) $$E_{T\left(30\right)}=\frac{28591}{\lambda {\left(RD\right)}_{max}}$$

(2) $$E_{T}^{N}=\frac{E_{T}\left(30\right)-30.7}{32.4}$$

(3) $$\pi^{*}=\frac{v{\left(DN\right)}_{max}-27.52}{-3.182}$$

(4) $$\alpha =\frac{E_{T}\left(30\right)-14.6\left(\pi^{*}-0.23\right)-30.31}{16.5}$$

(5) $$\beta =\frac{1.035v{\left(DN\right)}_{max}-v{\left(N\right)}_{max}+2.64}{2.8}$$

where: (RD)-Reichard’s dye, (*N*)-4-Nitroaniline, and (DN)-*N,N*-Diethyl-4-nitroaniline.

#### 2.2.3. Ionogels Samples Preparation

The samples were prepared in a glove box under a pure argon atmosphere. The photocurable composition consisted of a mixture of monomers, methacryl-POSS, solvents, and photoinitiator (0.2 wt.%) was homogenized in an orbital shaker. The obtained homogeneous composition was poured into 0.3 mm thick glass molds and exposed in (room temperature) RT to UV light for 10 min on each side of the mold (ASN-36W UV lamp, *λ_max_* = 365 nm, light intensity 6 mW∙cm^−2^, mold placed 4.5 cm from the light source). Then, the test samples were cut to certain dimensions from the obtained sheets of ionogels.

#### 2.2.4. Scanning Electron Microscope (SEM)

The studies of the morphology of the samples were performed with scanning electron microscope JEOL 7001F (SEI detector, 15 kV accelerating voltage). Before measurements, synthesized ionogels were treated with methanol and dried in an oven at 35 °C to remove the solvents mixture (IL + PC). The small pieces of dried samples were placed on a stub of metal with adhesive and coated with an ultrathin Gold/Palladium coating, deposited on the sample by low-vacuum sputter coating. Based on obtained photos, the range of diameters (nm) of polymer particles has been measured and means and standard deviations were calculated. To compare the obtained results, the statistical significance of the difference between means has been tested.

#### 2.2.5. Differential Scanning Calorimetry (DSC)

Glass transition temperature *T_g_* was measured by DSC (Mettler-Toledo DSC1 instrument, Greifensee, Switzerland) under a nitrogen atmosphere with a heating rate of 20 °C∙min^−1^ in the temperature range from −80 °C to 100 °C. The *T_g_* value was determined from the second run of the DSC measurement. For each ionogel, four measurements of DSC were made. Based on the obtained results, the means and standard deviations were calculated. To compare the obtained results, the statistical significance of the difference between means has been tested.

#### 2.2.6. TGA

Thermal resistance of synthesized materials was investigated with TG 209 F3 Tarsus thermogravimetric analyzer (NETZSCH-Geratebau GmbH, Norderstedt, Germany) in the temperature range of 30–600 °C. Approximately 10 mg of sample was placed in Al_2_O_3_ crucible and was analyzed with a heating rate of 10 °C∙min^−1^ under nitrogen atmosphere (purge of 10 mL∙min^−1^ of N_2_ protection gas and 20 mL∙min^−1^ of N_2_ sample gas).

#### 2.2.7. Puncture Resistance

To characterize the mechanical properties of the obtained ionogels, a puncture resistance test was conducted. The measurements were performed with a CT3 Texture Analyzer (Ametek Brookfield, Middleboro, MA, USA). A sample with a diameter of 16 mm was cut out from ionogel just after synthesis. Its thickness was measured and then the sample was fixed in a 10 mm hole diameter sample holder and tested for puncture strength using the spherical probe with 2.5 mm radius. During the measurement, the load and distance of the measuring probe were recorded until the sample was punctured (probe displacement rate–0.3 mm∙s^−1^). Because different ionogels slightly differ in their thickness, the load was normalized to a uniform thickness (300 µm) to compensate for the influence of thickness on the measurement. For each ionogel, seven measurements of mechanical properties were made. Based on the obtained results, the means and standard deviations were calculated. To compare the obtained results, the statistical significance of the difference between means has been tested.

#### 2.2.8. Ionic Conductivity

The ionic conductivity of the ionogels was investigated by electrochemical impedance spectroscopy (EIS) in the frequency range from 1 kHz to 1 MHz using the SP-300 potentiostat/galvanostat (Biologic, Seyssinet-Pariset, France). The experiment was performed in a two-electrode (stainless steel—316L) type electrochemical vessel at room temperature. The ionic conductivity of the ionogels (*σ*) was calculated from Equation (6):

(6) $$\sigma =\frac{l}{A\times \sigma_{s}}$$

where *σ* is the ionic conductivity of the ionogel in S∙cm^−1^, *l* is the thickness of the ionogel in cm, *A* is an ionogel surface area in cm^2^, and *σ_S_* represents the volumetric conductance of the ionogel sample, S.

For each ionogel, six measurements of conductivity were made. Based on the obtained results, the means and standard deviations were calculated. To compare the obtained results, the statistical significance of the difference between means has been tested.

#### 2.2.9. Electrochemical Measurements of Capacitors

##### Preparation of Electrodes

The carbon electrodes were prepared by the mixing of an appropriate amount of activated carbon (AC)—90 wt.% of Maxsorb MSP-20X (Kansai Coke and Chemicals CO., LTD., Hyogo, Japan)—with carbon black—5 wt.% of C65 (Imerys, Bironico Switzerland)—and binder—5 wt.% of PTFE (60 wt.% water suspension, Sigma–Aldrich, St. Louis, MO, USA). The electrode components were mixed in deionized water until a homogenous suspension was obtained. Then, the solvent was partially evaporated, and a thin film was prepared by rolling out and calendaring to an average thickness of 200 ± 15 μm. Thereafter, the electrode film was glued on a stainless steel current collector using Acheson electrodag PF-407C (Henkel, Dusseldorf, Germany). The mass and the diameter of each electrode were equal 13.0 ± 0.3 mg and 12 mm, respectively.

##### Electrochemical Investigations

Before assembling the Swagelok^®^ cell (Swagelok Switzerland, Wohlen, Switzerland), the carbon electrodes were soaked off in a solution of IL with PC, and the EC was assembled in an argon atmosphere. The cells were investigated by cyclic voltammetry (CV) with various scan rates from 5 to 100 mV∙s^−1^ and up to maximal cell potentials from 1 to 3 V, galvanostatic (0.5 to 4 A∙g^−1^) charge/discharge with potential limitation (GCPL), charge/discharge at constant power with voltage limitation between 1.5 to 3 V (CP, power in range 400 to 7000 Wh∙kg^−1^) and electrochemical impedance spectroscopy at OCV (EIS, over the frequency range from 1 MHz to 1 mHz with a 10 mV amplitude), using an SP-300 potentiostat/galvanostat (Biologic, France).

## 3. Results and Discussion 

**Figure 2:**
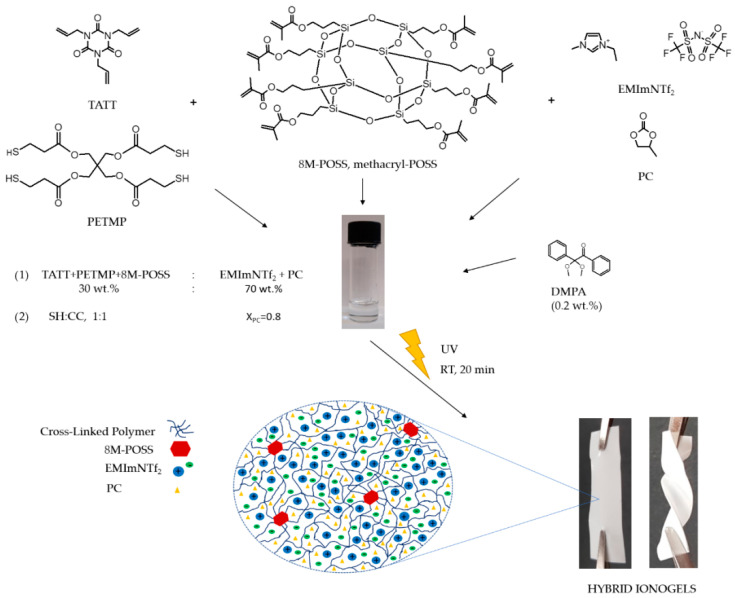
Schematic illustration of hybrid ionogels synthesis.

The thiol-ene system selected for this study was based on multifunctional monomers, i.e., trifunctional ene TATT and tetrafunctional thiol PETMP. Monomers were used in an equimolar ratio of functional groups (SH:C=C, 1:1). Polymerization of these monomers results in a high-density polymer network formed with a rapid reaction. However, the mechanical properties of the obtained polymer are insufficient due to the flexible thioether linkages formed during thiol-ene polymerization [34,35]. The presence of an IL in the ionogel based on this matrix causes an additional weakening of the mechanical properties. Thus, they require improvement. The introduction of methacryl-POSS (8M-POSS) containing a rigid core and eight reactive groups should improve the mechanical properties by the formation of a hybrid organic–inorganic thiol–ene-methacrylate network. As IL for ionogel preparation EMImNTf_2_ with high ionic conductivity (9 mS∙cm^−1^) and rather low sensitivity to moisture was used. However, considering the high viscosity and the price of ionic liquids, which make them not commonly used as electrolytes, we decided to prepare a mixture of IL with organic solvent—PC. This solvent is commonly used in electrochemistry [36,37]. The molar fraction of EMImNTf_2_ in PC was X = 0.2 (solution with the highest conductivity) and for ionogel preparation, 70 wt.% of this mixture was used. The introduction of propylene carbonate to the IL caused a significant increase in conductivity (13.8 mS∙cm^−1^); however, the use of this mixture for the synthesis of the ionogel caused leakage of the obtained material. For these two reasons, leakage and insufficient mechanical properties of the obtained ionogels, we decided to modify the polymer matrix by adding methacryl-POSS to the initial composition. With the addition of methacryl-POSS, an equimolar ratio of thiol (PETMP) to ene (TATT + 8M-POSS) functional groups was maintained, with the calculations assuming that 6 methacryl-POSS groups were involved in the polymerization reaction because of steric hindrance. The formulations of the compositions used for the synthesis of the polymer matrix are shown in Table 1. The hybrid ionogels preparation by one-pot reactions of TATT, PETMP, and 8M-POSS in existence with solvents mixture (EMImNTf_2_ and PC) is shown in Figure 2.

### 3.1. Photopolymerization Kinetics

Polymerization of thiol-ene compositions goes in a step-growth mechanism by hydrothiolation of double bonds. Polymerization is initiated by the decomposition of a photoinitiator and a hydrogen abstraction from a thiol functional group. Then chain propagation occurs in two steps: propagation reaction and chain-transfer reaction, which results in thiol addition across the alkene. This addition/chain-transfer process continues cyclically. The step-growth nature of this reaction requires an equimolar ratio of reactive groups SH:C=C (1:1) to obtain high conversion [38]. When methacrylate is added to thiol-ene composition or is used as ene monomer in this system, polymerization proceeds by homopolymerization of methacrylate–chain-growth reaction, and chain-transfer polymerization to thiol–step-growth polymerization [39]. In this way addition of methacryl-POSS to thiol-ene system will result in a hybrid (chain and step-growth) polymerization reaction.

**Figure 3:**
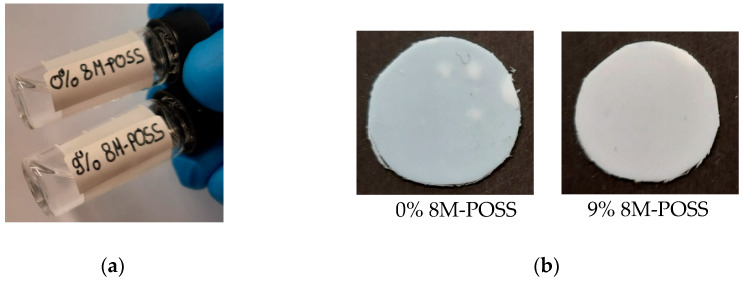
Optical images of (**a**) photocurable compositions in solvents mixture (70 wt.% of EMImNTf_2_ + PC): TATT + PETMP (0% 8M-POSS) and TATT + PETMP + 9 wt.% of 8M-POSS (9% 8M-POSS) and (**b**) ionogels poly(TATT + PETMP) and poly(TATT + PETMP + 9 wt.% of 8M-POSS) with 70 wt.% EMImNTf_2_ + PC.

Prepared initial compositions of monomers (TATT + PETMP) and modifier (8M-POSS) with mixture of solvents EMImNTf_2_ + PC were homogeneous and transparent (Figure 3a). After polymerization white, opaque ionogels were obtained (Figure 3b), which indicates phase separation between polymer matrix and solvents mixture. Phase separation was observed regardless of whether 8M-POSS was present in the investigated compositions. This phenomenon occurring during polymerization as well as the overlapping of absorption bands in the investigated compositions (C=C of triallyl isocyanurate with 8M-POSS, as well as double bonds with the absorption bands of the solvents mixture) and low intensities of the C=C and SH absorption bands (only 30 wt.% of monomers mixture in the composition) made it impossible to perform a kinetic study of thiol-ene photopolymerization by the infrared spectroscopy method. For this reason, the course of polymerization was investigated by the DSC method. Moreover, the triallyl monomer TATT can homopolymerize alongside the thiol-ene polymerization. This ability is rather small [38,40] however, we are unable to estimate it in solvents composition. Additional 8M-POSS is a methacrylate monomer that polymerizes in radical polymerization and homopolymerizes in thiol-ene polymerization to some extent. Thus, we expressed the reaction rate and conversions in the corresponding heat units (W∙g^−1^ and J∙g^−1^, resp.).

**Figure 4:**
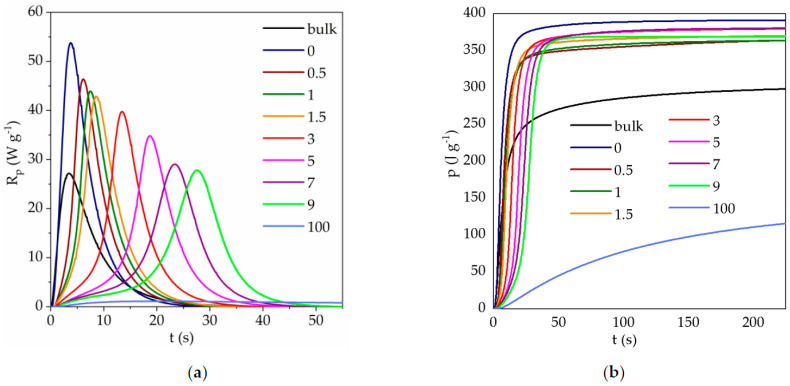
Photo-DSC kinetic curves (**a**) *R_p_ = f*(*t*), and (**b**) *p = f*(*t*) for 30 wt.% (TATT + PETMP + 8M-POSS) + 70 wt.% (EMImNTf_2_ + PC) system. Numbers indicate the 8M-POSS content in the matrix compositions expressed in wt.%, bulk—polymerization of TATT + PETMP composition without solvent.

**Figure 5:**
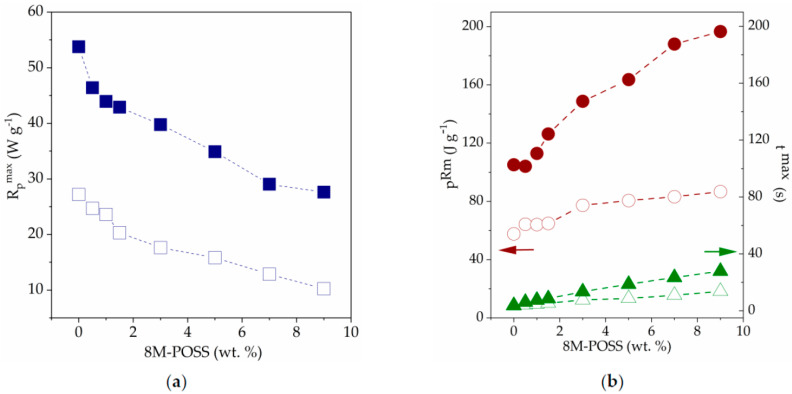
(**a**) Maximum polymerization rate (*R_p_^max^*), and (**b**) time at *R_p_^max^* (*t^ma^*^x^, triangles) and conversion at *R_p_^max^* (*p^Rm^*, circles) as a function of 8M-POSS content in investigated system (TATT + PETMP + 8M-POSS) with (full symbols) and without (open symbols) solvents mixture (EMImNTf_2_ + PC).

Kinetic curves obtained for photopolymerization of composition TATT + PETMP in the presence of 0, 0.5, 1, 1.5, 3, 5, 7, and 9 wt.% of 8M-POSS provided in 70 wt.% of solvents mixture (EMImNTf_2_ + PC) are depicted in Figure 4. Additionally, kinetic curve of 8M-POSS photopolymerization in 70 wt.% of the solvent mixture was performed (curve is designated as 100). The dependence of the maximum polymerization rate (*R_p_^max^*), time of reaction (*t^max^*), and conversion (*p^Rm^*) at *R_p_^max^* on the concentration of the 8M-POSS in the investigated systems are shown in Figure 5. 

The presented results indicate that the mixture of solvents EMImNTf_2_ and PC accelerate the thiol-ene polymerization of the TATT + PETMP system, but the addition of 8M-POSS causes a gradual decrease in the reaction rate and delays occurring of the maximum polymerization rate with increasing concentration of methacryl-POSS. 

The influence of ILs and non-ionic solvents on the thiol-ene polymerization we explain in our previous reports [11,41]. The accelerating effect of the solvent on thiol-ene polymerization we attribute to the polarity of the solvent, which can be described by the Kamlet–Taft solvent parameters. The parameters *α* and *β* are measures of hydrogen bond acidity (donating ability) and hydrogen bond basicity (accepting ability) of the solvent, respectively, and π* describe dipolarity/polarizability of the solvent [32,42]. The polar transition state for the reaction of a nucleophilic radical with a thiol is stabilized by polar solvents, which lowers the barrier for activation of hydrogen transfer [43]. The reaction proceeds faster in solvents with higher values of the *β* parameter which is connected with additional stabilization of the transition state during the chain-transfer step. On the other hand, the *α* parameter contributes to the decrease of polymerization rate due to the reduction in stabilization. In our previous study, we used monomer TATT and trimethylolpropane tris(3-mercaptopropionate) TMTMP, which differs only from the PETMP monomer in that it has one thiol group less in its structure. Thus, the influence of used solvents should be similar for the polymerization of these two compositions of monomers. Used solvents EMImNTf_2_ and PC caused the increase in polymerization rate of the TATT + TMTMP mixture, with the higher *R_p_*^max^ value obtained in PC. The IL EMImNTf_2_ has *β* parameter with lower value (0.23) than PC *β* parameter (0.35), and moreover with higher value of *α* parameter (*α*_EMImNTf2_ = 0.66, *α*_PC_ = 0.36). Therefore, the polymerization of TATT+TMTMP proceeds with a faster polymerization rate in the PC than in the IL. Thus, we expected in this study an increase in the polymerization rate using a mixture of PC and EMImNTf_2_ which is characterized by *α* Kamlet–Taft parameter equal to 0.59 and *β* parameter equal to 0.30. In addition, as can be seen in Figure 4, we observe a significant two-fold increase in the rate of polymerization of the TATT + PETMP composition in the presence of the solvent mixture EMImNTf_2_ and PC.

Solvent homopolymerization of the neat methacryl-POSS occurs with much lower reaction rate than solvent thiol-ene polymerization of the TATT + PETMP composition (Figure 4). The steric hindrance of POSS cages played important roles in preventing the homopolymerization of methacryl-POSS. Moreover, this monomer has eight reactive methacrylate groups per one molecule and polymerizes with the formation of a highly cross-linked polymer network and with immediate onset of the autoacceleration. This prevents the high conversion of the monomer.

The addition of methacryl-POSS modifier to thiol–ene-solvents composition causes a decrease in the polymerization rate of the TATT + PETMP system, which is related to the low homopolymerization rate of methacryl-POSS. The methacryl-POSS simultaneously participates in both reactions, homopolymerization of the double bond and chain-transfer to a thiol. As can be seen from Figure 4, methacrylate homopolymerization with a certain extent of chain-transfer to thiol dominates the initial stage of polymerization [44]. Then thiol-ene step-growth polymerization prevails. Such a course of polymerization also shifts the time (*t^max^*) and the conversion (*p^Rm^*) in the maximum reaction rate (*R_p_^max^*) to higher values (Figure 5). Additionally, methacryl-POSS causes a slight decrease in heat release during the polymerization course.

**Figure 6:**
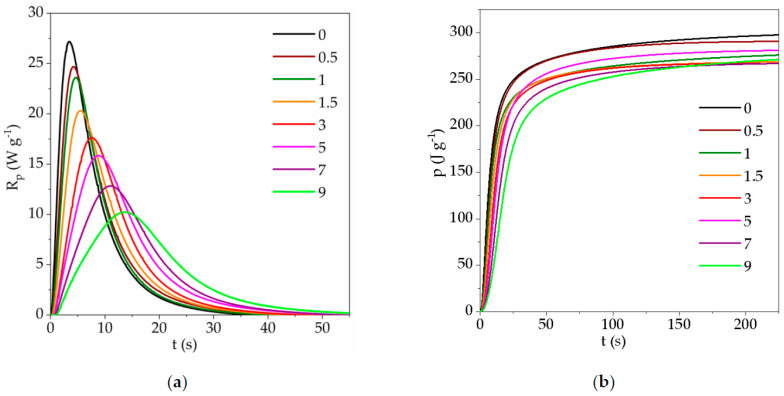
Photo-DSC kinetic curves (**a**) *R_p_ = f*(*t*), and (**b**) *p = f*(*t*) for TATT + PETMP + 8M-POSS system. Numbers indicate the 8M-POSS content in the compositions, expressed in wt.%.

We also performed investigations of the polymerization kinetics of thiol-en-8M-POSS systems in bulk (Figure 6), without the addition of a solvent mixture (EMImNTf_2_ + PC). As mentioned above, polymerization in bulk proceeds with a lower polymerization rate than in solvents mixture. As can be seen from the comparison of the obtained kinetic curves shown in Figure 4 and Figure 6, the addition of methacryl-POSS causes a decrease also in the rate of bulk polymerization, but the shapes of the kinetic curves are different than in the case of polymerization in the presence of solvents. The initial step associated with the homopolymerization of 8M-POSS is not so clearly marked. Bulk polymerization takes place in a system with a higher viscosity, and additionally, the lack of dilution of the system results in the formation of a polymer with a higher cross-link density.

### 3.2. Ionogels Morphology 

**Figure 7:**
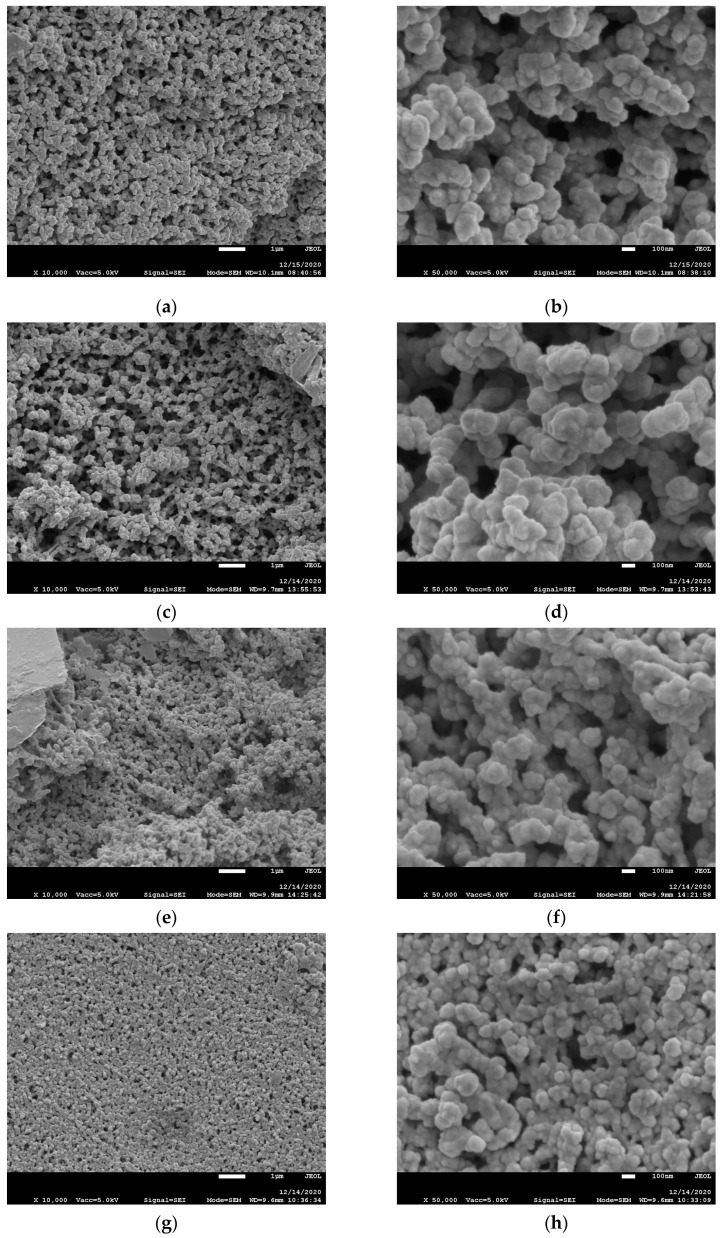
SEM images of ionogels matrices prepared without addition of 8M-POSS (**a**,**b**) and in the presence of 1.5 wt.% (**c**,**d**), 5 wt.% (**e**,**f**), and 7 wt.% (**g**,**h**) of 8M-POSS. (Magnification: (**a**,**c**,**e**,**g**)—10,000×, and (**b**,**d**,**f**,**h**)—50,000×).

As was mentioned above obtained ionogels were opaque, with phase separation. Thus, the polymer matrix morphology of synthesized ionogels, after extraction of solvents, was investigated by SEM to reveal the structure of the polymer matrix. SEM images of polymer matrices are shown in Figure 7. Polymer matrices with and without 8M-POSS modification show morphology of connected spheres which exhibit rather uniform dimensions. This morphology indicates that thiol-ene polymerization proceeds as a dispersion polymerization. In this type of polymerization, the size of polymer particles and their distribution is affected by many parameters, such as the medium, type and concentration of monomer, stabilizer and initiator, and the temperature of the process. Moreover, monomer, stabilizer, and initiator should be soluble and polymer insoluble in the medium. Additionally, the stabilizer should deposit on the surface of the particles for their stabilization. We obtained monodisperse microspheres that indicate that nucleation takes place in the initial stage of polymerization, at low monomer conversion. Polymer precipitates in dispersion polymerization when it reaches a critical length/size forming the nuclei. These particle nuclei farther react with growing polymeric chains which leads to uniformly growing microspheres. Monodispersity is considered to take place when the particle growth step is long in relation to the nucleation step [45]. In our previous studies, we also obtained ionogels with the morphology of the connected spheres which we attributed to the stabilizing effect of the IL [11]. In this study, the mixture of IL and PC was used so the ionic liquid content in the solvent is lower. As a result, the spheres do not have such distinct shapes and coagulate to a greater extent than in the case of thiol-ene synthesis provided only in an IL. The stabilization effect of IL in PC is smaller than in the case of neat IL. The addition of PC to IL changes the solvency of the medium, which is one of the most important factors affecting particle formation in dispersion polymerization. 

As can be seen in Figure 7, the addition of 8M-POSS does not change the morphology but does affect the polymer particle size. Therefore, the size of the polymer spheres was measured, and statistical calculations were performed. The obtained results are presented in Table 2. The higher is the concentration of methacryl-POSS in the starting composition, the smaller are the dimensions of the formed polymer spheres. The introduction of POSS to the composition affects the kinetics of thiol-ene polymerization, causing a decrease in the reaction rate due to the greater share of methacrylate polymerization in the initial stage of the process. Methacryl-POSS has eight reactive methacrylate groups per molecule so with increasing concentration of this monomer, the functionality of polymerizing medium increases. In addition, with an increasing number of functional groups, the microsphere diameter decreases because the polymer has poorer solubility in the polymerizing medium at a similar conversion, and nucleation begins at lower conversions. Thus, more nuclei are formed [46]. For all investigated systems, a similar final conversion is achieved (similar heat release, Figure 4b), so for each formed nucleus, a lower number of monomer molecules are attached during the growth stage of the particle. And as a result, smaller microspheres are formed.

### 3.3. Thermal and Mechanical Properties

#### 3.3.1. Thermal Properties

The glass transition temperatures *T_g_* of synthesized materials were measured using DSC. In Table 3 the *T_g_* temperatures of the polymer matrix, ionogel without modification, and hybrid ionogels are presented. Pure poly(TATT+PETMP) has glass transition above room temperature, i.e., *T_g_* = 35.1 °C because two multifunctional monomers are used to matrix synthesis what results in a dense polymer network formation. Glass transition decreases about 20 °C for ionogel with 70 wt.% of EMImNTf_2_ + PC mixture. Used solvents plasticize polymer matrix. As shown in Table 3, the addition of methacryl-POSS to the polymerizing compositions caused a slight increase of *T_g_* of hybrid ionogels of about 1–3 °C. However, in the studied range of 8M-POSS concentration, considering the results of the statistical analysis for this parameter (Table S2, Supplementary Materials), no dependence of *T_g_* on the 8M-POSS concentration in ionogel is observed. It seems that there are several reasons for this slight change in the *T_g_* values of the investigated ionogels. On the one hand, the presence of the rigid inorganic core and eight reactive groups in the methacryl-POSS molecule should contribute to the increase of glass temperature of ionogels. On the other hand, the lower conversion of SH groups (due to the homopolymerization of 8M-POSS), and thus the presence of unreacted thiol groups in the matrix structure, may cause a plasticizing effect (decrease of *T_g_*). The additional effect can be exerted by the intermolecular interactions of the polymer matrix with the solvent mixture. Stronger for modified hybrid ionogels.

**Figure 8:**
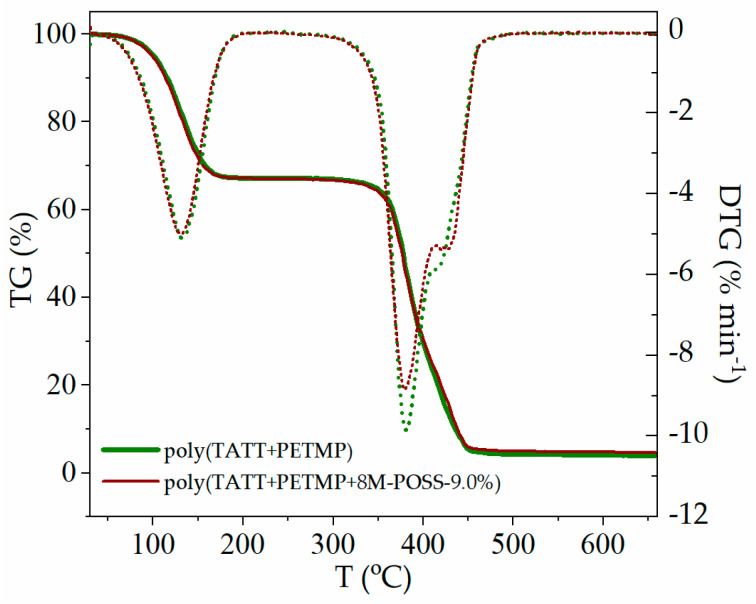
TG and DTG curves of ionogels with 70 wt.% of EMImNTf_2_ and PC mixture. TG—solid line, DTG—dotted line.

Thermal gravimetric analysis was used to investigate the thermal stabilities of synthesized ionogels. Figure 8 shows the thermogravimetric (TG) curves and derivative thermogravimetric (DTG) curves of two types of ionogels, neat ionogel and hybrid ionogel modified with 9 wt.% of methacryl-POSS. The analyzed data are summarized in Table 3. Temperatures at 5% (*T*_5%_) and 10% (*T*_10%_) of weight loss represent the onset temperature of the material decomposing [35].

Figure 8 shows the weight loss of the materials when heated up to 600 °C under a nitrogen atmosphere. As can be seen, synthesized ionogels possess three steps of decomposition. The first one, occurring at a temperature of about 100 °C, can be attributed to the evaporation of one of the solvents, i.e., propylene carbonate. The next one is the weight loss of the polymer matrix, and the last one is related to IL decomposition. Polymer matrix without a solvent decomposes in one stage. The presence of methacryl-POSS in the polymer matrix of ionogels does not actually affect the temperature of the initial decomposition. 

#### 3.3.2. Mechanical Properties

Flexible and quite mechanically strong materials as for gels were obtained, i.e., ionogels can be twisted or rolled-up without damaging it. Their mechanical properties were investigated as puncture resistance. As can be seen in Table 4 the puncture strength (*F_max_*) of synthesized ionogel poly(TATT + PETMP) with 70 wt.% of IL + PC is equal to 247 g. Modification with methacryl-POSS caused an increase in the values of this parameter. The addition of methacryl-POSS in the amount of up to 1.5–5.0 wt.% causes an increase in the parameter with an increase in modifier concentration, reaching the maximum value of ca. 330 g—there is no statistically significant differences in puncture strength for ionogels containing 1.5–5 wt.% of methacryl-POSS (results of the post hoc test are presented in Table S3 in Supplementary Materials). Further increase in the modifier content causes a decrease in the puncture strength. The increase in mechanical properties is resultant from the increased cross-linking density and incorporation of the rigid core of the POSS cage into the polymer network. Deterioration of mechanical properties at higher POSS contents may be associated with a decrease in the size of the polymer particles obtained in dispersion polymerization. The addition of methacryl-POSS has no effect on elongation at break (*ε_max_*).

### 3.4. Electrochemical Investigation

#### 3.4.1. Ionic Conductivity

**Figure 9:**
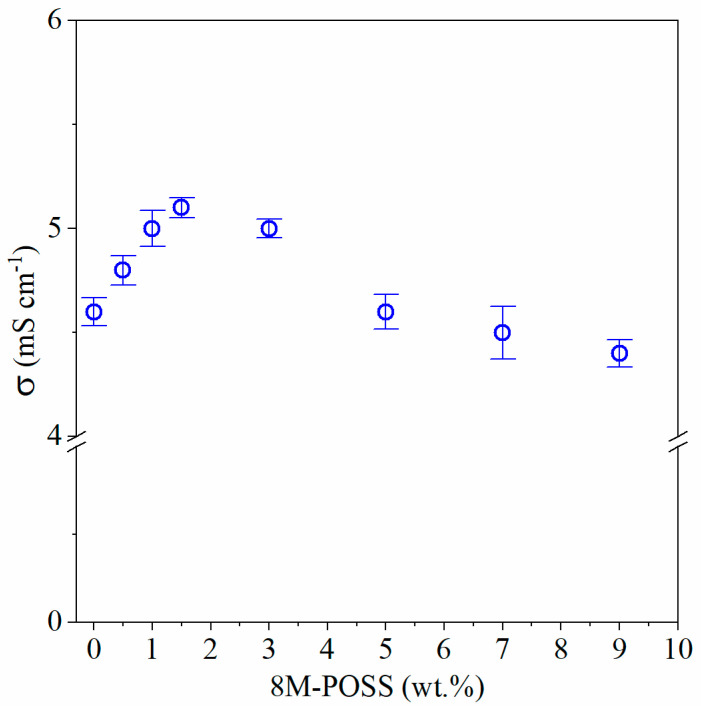
Ionic conductivity (with standard deviation) of ionogels modified with different amounts of methacryl-POSS.

Next to mechanical properties, ionic conductivity is an important parameter that decides the utility of ionogels as GPE in EC. In Figure 9 the dependence of ionic conductivity on methacryl-POSS concentration in the synthesized ionogels is presented. The ionic conductivity slightly increases with increasing concentration of methacryl-POSS in ionogel reaching ca. 5.0 mS∙cm^−1^ at methacryl-POSS content equal 1–3 wt.%—there is no statistically significant differences in ionic conductivity for ionogels containing 1–3 wt.% of methacryl-POSS (results of the post hoc test are presented in Table S4 in Supplementary). Further increasing of 8M-POSS concentration causes decreasing of conductivity, achieving 4.4 mS∙cm^−1^ at 8M-POSS content equal to 9 wt.%. Presented results clearly show that low content of methacryl-POSS in ionogel can slightly improve (ca. 10%) ionic conductivity. This observation can be explained by the promotion of ion-pair dissociations in IL clusters caused by methacryl-POSS [24].

#### 3.4.2. Electrochemical Capacitor Investigation

Considering mechanical properties (Table 4) and ionic conductivity (Figure 9) it seems that ionogels with methacryl-POSS content in the range 1.5–3 wt.% (highest ionic conductivity and mechanical properties at the same time) are the most valuable for their application as GPE in EC. Thus, we decided to construct EC with ionogels containing 1.5 wt.% and 0 wt.% (for comparison) of 8M-POSS.

**Figure 10:**
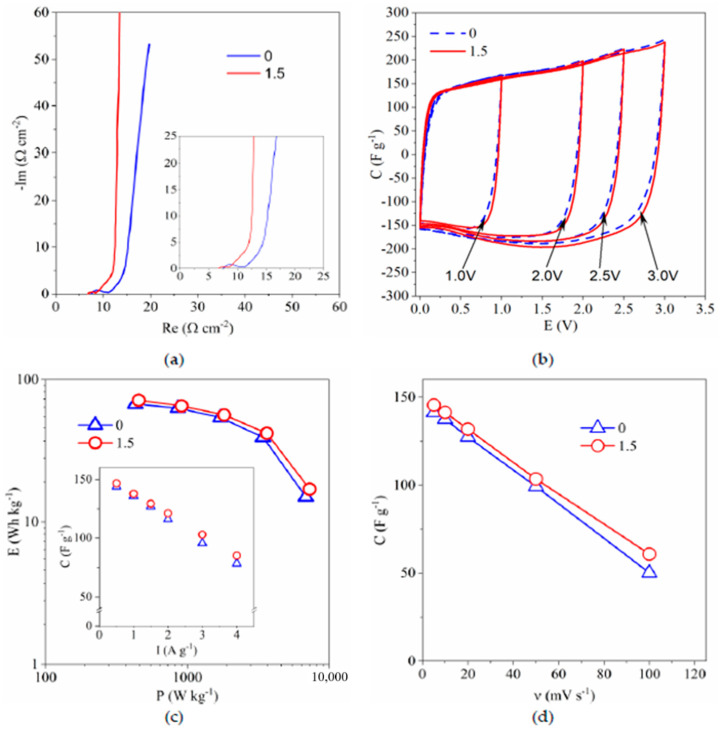
(**a**) Nyquist plot, (**b**) Cyclic voltammograms up to various values of maximum potential. Scan rate 5 mV∙s^−1^, (**c**) Ragone plot and discharge capacitance vs current (inset) of the ECs, (**d**) discharge capacitance vs scan rate of AC/AC capacitors with applied ionogels. Capacitance, current, energy, and power expressed per AC mass in one electrode. Numbers indicate the 8M-POSS content in the compositions expressed in wt.%.

The Nyquist plot of the AC/AC capacitor (Figure 10a) equivalent series resistance (ESR) equal ca. 7.5 Ω and 6.8 Ω for 0 wt.% and 1.5 wt.% of 8M-POSS, respectively. These values are slightly higher than the resistance of ionogels (5.8 Ω and 5.2 Ω for 0 wt.% and 1.5 wt.% of methacryl-POSS respectively) applied in EC. We can also notice that difference in ESR value corresponds very well to the difference in resistance of ionogels. It shows that most of the resistance relates to the resistance of ionogel. Nevertheless, total EC resistance is not high, and we can observe good electrochemical properties of the device (Figure 10b–d).

The CV of the AC/AC capacitor (Figure 10b) obtained at voltages up to 3.0 V is close to the box-like shape of an electric double-layer capacitor (EDLC). The absence of peaks on the CV curve exhibits a capacitive behavior of EDLC without redox reaction. In Figure 10c (inset) the discharge capacitance vs current is presented. As it can be seen, current changing from 0.5 A∙g^−1^ to 4 A∙g^−1^ causes linear decreasing of capacitance from ca. 144 F∙g^−1^ to ca. 78 F∙g^−1^ and from ca. 147 F∙g^−1^ to ca. 88 F∙g^−1^ for EC with ionogel containing 0 wt.% and 1.5 wt.% of methacryl-POSS. Similar behavior can be observed in the dependence of capacitance vs. scan rate (Figure 10d), where linear decreasing of capacitance can be seen. Also, the Ragone plot (Figure 10c) presents very good properties of investigated EC. Increasing power density from ca. 430 W∙kg^−1^ to ca. 3400 W∙kg^−1^ (0 wt.% of methacryl-POSS) and from ca. 460 W∙kg^−1^ to ca. 3600 W∙kg^−1^ (1.5 wt.% of methacryl-POSS), causes only a slight decrease of energy density from ca. 67 W∙kg^−1^ to ca. 40 W∙kg^−1^ and from ca. 72 W∙kg^−1^ to ca. 42 W∙kg^−1^ for 0 wt.% and 1.5 wt.% of methacryl-POSS, respectively. Comparing the results obtained for capacitors with both ionogels, we can clearly see that ECs present similar capacitance/energy at a low value of current density (I), scan rate (*v)*, or power (P). Thus, with increasing of these parameters (I, v, P) we can observe the greater difference in obtained values of capacitance and energy, with better results obtained for EC with ionogel containing 1.5 wt.% of methacryl-POSS. Furthermore, we should consider that both ionogels have the same thickness, and the ionogel containing 1.5 wt.% of methacryl-POSS shows better mechanical properties by ca. 30% at the same time. If we would like to apply a modified ionogel with similar mechanical properties as unmodified, we can reduce the thickness by about 30% and likewise reduce the resistance of the modified ionogel by about 30% (1.5 Ω). This will allow us to further improve the electrochemical properties of the EC.

All these results show that modification of ionogels by 8M-POSS modifier and its application as GPE in electrochemical capacitor allows obtaining EC with very good electrochemical properties.

## 4. Conclusions

The hybrid ionogels modified with methacryl-POSS were successfully prepared by thiol-ene photopolymerization in a mixture of the imidazolium IL EMImNTf_2_ and PC. Solvents mixture accelerates the thiol-ene polymerization of the TATT + PETMP system which was attributed to the polarity of the solvent which lowers the barrier for activation of hydrogen transfer in thiol-ene polymerization. On the other hand, methacryl-POSS decreases thiol-ene polymerization rate because of the low homopolymerization rate of methacryl-POSS.

The main disadvantage of the unmodified ionogel is leakage which makes the ionogel impossible to use as a gel polymer electrolyte. However, the modification with methacryl-POSS made it possible to obtain ionogels with no leakage. In addition, methacryl-POSS contributed to the improvement of the mechanical properties of hybrid ionogels. An increase in puncture strength (by approx. 30%) was observed, as well as a slight increase in the glass transition temperature. This is related to the steric hindrance of the POSS cages as well as the increase of cross-linking density of the polymer matrix. Additionally, the size of the interconnected polymer microspheres formed in dispersion polymerization decreases with the increase of methacryl-POSS content. The reason is poorer solubility of the modified polymer in the polymerizing medium at a similar conversion, so nucleation begins at lower conversions. Modification of ionogels with methacryl-POSS also causes an increase in ionic conductivity, by the promotion of ion-pair dissociations in IL clusters caused by methacryl-POSS. Moreover, application of hybrid ionogel as an GPE in an electrochemical capacitor allows obtaining EC with very good electrochemical properties.

## Figures and Tables

**Table 1:** Formulations of the thiol-ene matrix with 0.2 wt.% of DMPA photoinitiator.

Formulation	PETMP	TATT	8M-POSS
	wt.%		
8M-POSS-0%	59.5	40.5	0
8M-POSS-0.5%	59.3	40.2	0.5
8M-POSS-1.0%	59.1	39.9	1.0
8M-POSS-1.5%	58.9	39.6	1.5
8M-POSS-3.0%	58.4	38.6	3.0
8M-POSS-5.0%	57.6	37.4	5.0
8M-POSS-7.0%	56.8	36.2	7.0
8M-POSS-9.0%	56.0	35.0	9.0

**Table 2:** The **r**ange of diameters (nm)of polymer particles formed in EMImNTf_2_ + PC mixture.

Formulation	Particle Size, nm
8M-POSS-0%	165 ± 15
8M-POSS-0.5%	141 ± 13
8M-POSS-1.5%	128 ± 12
8M-POSS-5.0%	60 ± 6.1
8M-POSS-7.0%	46 ± 4.5

Results of the post hoc test are presented in Table S1 in Supplementary Materials.

**Table 3:** Thermal properties of synthesized materials.

Investigated Materials		T_g_, °C	T_5%_, °C	T_10%_, °C
matrix	poly(TATT+PETMP)	35.1 ± 0.77	360.2	368.3
ionogels poly(TATT + PETMP + 8M-POSS) with 70 wt.% of EMImNTf_2_ + PC	8M-POSS-0%	13.4 ± 1.1	100.1	115.1
	8M-POSS-0.5%	14.2 ± 1.2	103.5	117.6
	8M-POSS-1.0%	15.3 ± 0.75	103.1	117.1
	8M-POSS-1.5%	16.3 ± 0.60	101.0	116.2
	8M-POSS-3.0%	16.7 ± 0.79	98.2	112.8
	8M-POSS-5.0%	16.0 ± 0.74	100.2	114.6
	8M-POSS-7.0%	16.1 ± 0.50	100.1	114.7
	8M-POSS-9.0%	16.5 ± 0.42	99.4	113.9

**Table 4:** Mechanical properties of ionogels with 70 wt.% of EMImNTf_2_ + PC.

Formulation	*F_max_*, g	*ε_max_*, mm
8M-POSS-0%	247 ± 9.1	4.1 ± 0.21
8M-POSS-0.5%	304 ± 12	4.3 ± 0.08
8M-POSS-1.0%	308 ± 3.9	4.1 ± 0.13
8M-POSS-1.5%	322 ± 6.4	4.2 ± 0.15
8M-POSS-3.0%	331 ± 7.6	4.3 ± 0.15
8M-POSS-5.0%	336 ± 14	4.4 ± 0.14
8M-POSS-7.0%	305 ± 6.4	4.0 ± 0.07
8M-POSS-9.0%	264 ± 8.0	3.9 ± 0.09

## Supplementary Materials

The following are available online at https://www.mdpi.com/2073-4360/13/3/385/s1, Table S1. Statistical significance analysis of the diameters of polymer particles formed in EMImNTf2+ PC mixture. *p*-values of post hoc test—multiple comparisons of mean ranks (Dunn’s test). Values of *p* < 0.05 are marked in red; Table S2. Statistical significance analysis of the glass transition temperatures *T_g_* of synthesized materials. *p*-values of post hoc test—Tukey HSD method. Values of *p* < 0.05 are marked in red; Table S3. Statistical significance analysis of the mechanical properties of ionogels. *p*-values of post hoc test—Tukey HSD method. Values of *p* < 0.05 are marked in red; Table S4. Statistical significance analysis of the ionic conductivity of ionogels. *p*-values of post hoc test—Tukey HSD method. Values of *p* < 0.05 are marked in red.
