# Supplementary material for: Modification of Thiol-Ene Ionogels with Octakis(methacryloxypropyl) Silsesquioxane
Source: Polymers (Basel). 2021 Jan 26;13(3):385. doi: 10.3390/polym13030385 (PMC7865288; doi:10.3390/polym13030385)
Supplement: Supplementary file 1 [file polymers-13-00385-s001.pdf]

polymers

MDPI

Supplementary

Modification of Thiol–Ene Ionogels with Octakis(methacryloxypropyl) Silsesquioxane

Aneta Lewandowska, Piotr Gajewski, Katarzyna Szcześniak, Mariola Sadej, Piotr Patelski and Agnieszka Marcinkowska *

Poznan University of Technology, Institute of Chemical Technology and Engineering, Faculty of Chemical Technology, Department of Polymers, Berdychowo 4, 60-965 Poznan, Poland; aneta.b.lewandowska@doctorate.put.poznan.pl (A.L); piotr.gajewski@put.poznan.pl (P.G) katarzyna.szczesniak@put.poznan.pl (K.S); mariola.sadej@put.poznan.pl (M.S); piotr.r.patelski@student.put.poznan.pl (P.P)

* Correspondence: agnieszka.marcinkowska@put.poznan.pl (A.M); Tel.: +48616653637

Table S1 presents the p-values of post hoc test results of statistical significance analysis of the diameters of polymer particles formed in $EMImNTf_2$+PC mixture. Because the assumption of homogeneity of variance is not met, the multiple comparisons of mean ranks (Dunn's test) were used. As it can be seen, almost all *p*-values are less than the significance level (α=0.05), what suggests that the diameters of polymer particles significantly differ from each other.

Table S1. Statistical significance analysis of the diameters of polymer particles formed in EMImNTf2+ PC mixture. *p*-values of post hoc test—multiple comparisons of mean ranks (Dunn's test). Values of p<0.05 are marked in red.

Formulation	POSS-0%	POSS-0.5%	POSS-1.5%	POSS-5.0%	POSS-7.0%
POSS-0%		5.0×10^{-2}	1.2×10^{-4}	0.0×10^{0}	0.0×10^{0}
POSS-0.5%	5.0×10^{-2}		1.0×10^{0}	4.6×10^{-6}	0.0×10^{0}
POSS-1.5%	1.2×10^{-4}	1.0×10^{0}		5.3×10^{-3}	3.0×10^{-8}
POSS-5.0%	0.0×10^{0}	4.6×10^{-6}	5.3×10^{-3}		4.8×10^{-2}
POSS-7.0%	0.0×10^{0}	0.0×10^{0}	3.0×10^{-8}	4.8×10^{-2}	

Table S2 presents the *p*-values of post hoc test (Tukey HSD method) results of statistical significance analysis of the glass transition temperatures T_g of synthesized materials. As it can be seen, the T_g values of the polymer matrix and polymer matrix with $EMImNTf_2$+PC mixture differs significantly from each other and from 8M-POSS modified ionogels. At the same time, for all 8M-POSS modified ionogels the difference of T_g values is statistically insignificant.

Table S2. Statistical significance analysis of the glass transition temperatures T_g of synthesized materials. *p*-values of post hoc test—Tukey HSD method. Values of p<0.05 are marked in red.

Formulation	p(TATT+PETM)	POSS-0%	POSS-0.5%	POSS-1.0%	POSS-1.5%	POSS-3.0%	POSS-5.0%	POSS-7.0%	POSS-9.0%
p(TATT+PETM)		1.4×10^{-4}	1.4×10^{-4}	1.4×10^{-4}	1.4×10^{-4}	1.4×10^{-4}	1.4×10^{-4}	1.4×10^{-4}	1.4×10^{-4}
POSS-0%	1.4×10^{-4}		2.1×10^{-2}	8.0×10^{-4}	2.0×10^{-4}	1.8×10^{-4}	1.4×10^{-4}	2.9×10^{-4}	1.5×10^{-4}
POSS-0.5%	1.4×10^{-4}	2.1×10^{-2}		9.7×10^{-1}	6.9×10^{-1}	6.3×10^{-1}	2.9×10^{-1}	8.4×10^{-1}	3.9×10^{-1}
POSS-1.0%	1.4×10^{-4}	8.0×10^{-4}	9.7×10^{-1}		1.0×10^{0}	1.0×10^{0}	9.2×10^{-1}	1.0×10^{0}	9.6×10^{-1}
POSS-1.5%	1.4×10^{-4}	2.0×10^{-4}	6.9×10^{-1}	1.0×10^{0}		1.0×10^{0}	1.0×10^{0}	1.0×10^{0}	1.0×10^{0}
POSS-3.0%	1.4×10^{-4}	1.8×10^{-4}	6.3×10^{-1}	1.0×10^{0}	1.0×10^{0}		1.0×10^{0}	1.0×10^{0}	1.0×10^{0}
POSS-5.0%	1.4×10^{-4}	1.4×10^{-4}	2.9×10^{-1}	9.2×10^{-1}	1.0×10^{0}	1.0×10^{0}		9.9×10^{-1}	1.0×10^{0}
POSS-7.0%	1.4×10^{-4}	2.9×10^{-4}	8.4×10^{-1}	1.0×10^{0}	1.0×10^{0}	1.0×10^{0}	9.9×10^{-1}		1.0×10^{0}
POSS-9.0%	1.4×10^{-4}	1.5×10^{-4}	3.9×10^{-1}	9.6×10^{-1}	1.0×10^{0}	1.0×10^{0}	1.0×10^{0}	1.0×10^{0}	

Table S3 presents the *p*-values of post hoc test (Tukey HSD method) results of statistical significance analysis of the mechanical properties of ionogels. As the analysis shows,

there are no statistically significant differences in mechanical properties between iongels containing 1.5 to 5 wt. % of methacryl-POSS.

Table S3. Statistical significance analysis of the mechanical properties of ionogels. *p*-values of post hoc test—Tukey HSD method. Values of $p<0.05$ are marked in red.

Formulation	POSS-0%	POSS-0.5%	POSS-1.0%	POSS-1.5%	POSS-3.0%	POSS-5.0%	POSS-7.0%	POSS-9.0%
POSS-0%		1.4×10^{-4}	1.4×10^{-4}	1.4×10^{-4}	1.4×10^{-4}	1.4×10^{-4}	1.4×10^{-4}	1.6×10^{-2}
POSS-0.5%	1.4×10^{-4}		9.9×10^{-1}	3.8×10^{-2}	2.1×10^{-4}	1.4×10^{-4}	1.0×10^{0}	1.4×10^{-4}
POSS-1.0%	1.4×10^{-4}	9.9×10^{-1}		2.6×10^{-1}	1.7×10^{-3}	2.3×10^{-4}	9.9×10^{-1}	1.4×10^{-4}
POSS-1.5%	1.4×10^{-4}	3.8×10^{-2}	2.6×10^{-1}		4.9×10^{-1}	9.8×10^{-2}	5.2×10^{-2}	1.4×10^{-4}
POSS-3.0%	1.4×10^{-4}	2.1×10^{-4}	1.7×10^{-3}	4.9×10^{-1}		9.8×10^{-1}	2.8×10^{-4}	1.4×10^{-4}
POSS-5.0%	1.4×10^{-4}	1.4×10^{-4}	2.3×10^{-4}	9.8×10^{-2}	9.8×10^{-1}		1.4×10^{-4}	1.4×10^{-4}
POSS-7.0%	1.4×10^{-4}	1.0×10^{0}	9.9×10^{-1}	5.2×10^{-2}	2.8×10^{-4}	1.4×10^{-4}		1.4×10^{-4}
POSS-9.0%	1.6×10^{-2}	1.4×10^{-4}	1.4×10^{-4}	1.4×10^{-4}	1.4×10^{-4}	1.4×10^{-4}	1.4×10^{-4}	

Table S4 presents the *p*–values of post hoc test (Tukey HSD method) results of statistical significance analysis of the ionic conductivity of ionogels. As the analysis shows, there are no statistically significant differences in the ionic conductivity between ionogels containing 1.0 to 3.0 wt.% of methacryl–POSS.

Table S4. Statistical significance analysis of the ionic conductivity of ionogels. *p*–values of post hoc test—Tukey HSD method. Values of $p<0.05$ are marked in red.

Formulation	POSS–0%	POSS–0.5%	POSS–1.0%	POSS–1.5%	POSS–3.0%	POSS–5.0%	POSS–7.0%	POSS–9.0%
POSS-0%		1.6×10^{-2}	1.5×10^{-4}	1.5×10^{-4}	1.5×10^{-4}	1.0×10^{0}	9.6×10^{-1}	3.8×10^{-2}
POSS-0.5%	1.6×10^{-2}		4.3×10^{-2}	4.3×10^{-4}	2.5×10^{-3}	1.2×10^{-2}	1.4×10^{-3}	1.5×10^{-4}
POSS-1.0%	1.5×10^{-4}	4.3×10^{-2}		4.7×10^{-1}	9.1×10^{-1}	1.5×10^{-4}	1.5×10^{-4}	1.5×10^{-4}
POSS-1.5%	1.5×10^{-4}	4.3×10^{-4}	4.7×10^{-1}		9.9×10^{-1}	1.5×10^{-4}	1.5×10^{-4}	1.5×10^{-4}
POSS-3.0%	1.5×10^{-4}	2.5×10^{-3}	9.1×10^{-1}	9.9×10^{-1}		1.5×10^{-4}	1.5×10^{-4}	1.5×10^{-4}
POSS-5.0%	1.0×10^{0}	1.2×10^{-2}	1.5×10^{-4}	1.5×10^{-4}	1.5×10^{-4}		9.8×10^{-1}	5.2×10^{-2}
POSS-7.0%	9.6×10^{-1}	1.4×10^{-3}	1.5×10^{-4}	1.5×10^{-4}	1.5×10^{-4}	9.8×10^{-1}		2.9×10^{-1}
POSS-9.0%	3.8×10^{-2}	1.5×10^{-4}	1.5×10^{-4}	1.5×10^{-4}	1.5×10^{-4}	5.2×10^{-2}	2.9×10^{-1}	
